# Ophthalmologic Manifestations in Bardet–Biedl Syndrome: Emerging Therapeutic Approaches

**DOI:** 10.3390/medicina61071135

**Published:** 2025-06-24

**Authors:** Amaris Rosado, Ediel Rodriguez, Natalio Izquierdo

**Affiliations:** 1 School of Medicine, Medical Sciences Campus, University of Puerto Rico, San Juan 00936-5067, Puerto Rico; ediel.rodriguez@upr.edu; 2 Department of Ophthalmology, Medical Sciences Campus, University of Puerto Rico, San Juan 00936-5067, Puerto Rico; njuan@msn.com

**Keywords:** Bardet–Biedl syndrome, retinal degeneration, rod–cone dystrophy, ciliopathy, photoreceptor degeneration, gene therapy

## Abstract

Bardet–Biedl syndrome (BBS) is a rare multisystem ciliopathy characterized by early-onset retinal degeneration and other vision-threatening ophthalmologic manifestations. This review synthesizes current knowledge on the ocular phenotype of BBS as well as emerging therapeutic approaches aimed at preserving visual function. Retinal degeneration, particularly early macular involvement and rod–cone dystrophy, remains the hallmark of BBS-related vision loss. Additional ocular manifestations, such as refractive errors, nystagmus, optic nerve abnormalities, and cataracts further contribute to visual morbidity. Experimental therapies—including gene-based interventions and pharmacologic strategies such as nonsense suppression and antioxidant approaches—have shown promise in preclinical models but require further validation. Early ophthalmologic care, including routine visual assessments, refractive correction, and low-vision rehabilitation, remains the standard of management. However, there are currently no effective therapies to halt or reverse retinal degeneration, which underscores the importance of emerging molecular and genetic interventions. Timely recognition and comprehensive ophthalmologic evaluation are essential to mitigate visual decline in BBS. Future efforts should focus on translating these approaches into clinical practice, enhancing early diagnosis, and promoting multidisciplinary collaboration to improve long-term outcomes for patients with BBS.

## 1. Introduction

Ciliopathies are a group of disorders caused by impaired function of the primary cilium, resulting in a wide spectrum of clinical manifestations. Cilia are broadly classified into two types: motile and non-motile (primary). Motile cilia are mainly involved in fluid transport, cell motility, and clearing particles across epithelial surfaces. In contrast, primary cilia function as sensory organelles that mediate intracellular signaling essential for normal tissue development and homeostasis [1,2]. These cilia regulate key signaling pathways such as Hedgehog, Wnt, Notch, and mTOR, which are critical for cell polarity, differentiation, and proliferation [3,4]. Dysfunction of primary cilia can lead to isolated organ disease or complex multisystem syndromes, such as BBS, which may include retinal degeneration, renal anomalies, polydactyly, and obesity [5].

BBS is a rare inherited disorder classified as a primary ciliopathy. It is caused by autosomal recessive mutations in genes critical for ciliary structure and function. To date, 27 genes have been identified as causative, most of which encode components of the BBSome complex, chaperonin-like proteins, or elements involved in intraflagellar transport (IFT) [6,7]. The most frequently implicated genes are BBS1 and BBS10, accounting for the majority of molecularly confirmed cases [8,9].

The incidence of BBS is estimated to be between 1 in 120,000 and 160,000 in the United States and Europe, with significantly higher rates reported in genetically isolated populations [8,9]. Due to its genetic pleiotropy and high heterogeneity, BBS presents with a wide range of phenotypic features. Diagnosis is typically based on the presence of either four major features or three major plus two minor features [10]. Major manifestations include rod–cone dystrophy, truncal obesity, postaxial polydactyly, cognitive impairment, learning difficulties, hypogonadism, renal anomalies, and genitourinary abnormalities. Minor features consist of diabetes mellitus, ataxia, hypertonia, oral and dental abnormalities, congenital heart defects, hepatic fibrosis, facial dysmorphism, digital anomalies such as syndactyly, and impaired olfaction [6].

As with other syndromic ciliopathies, the most common and consistent feature in BBS is retinal rod–cone dystrophy, which affects both peripheral and central vision [11,12]. Despite its early onset, the diagnosis of BBS is frequently delayed and often made only after ophthalmologic evaluation [6]. In many cases, symptoms of retinal dystrophy, such as night blindness (nyctalopia), hyperopic astigmatism, ptosis, or mild blepharospasm during early childhood are the initial reasons for seeking medical care [8,13,14]. The condition progresses slowly but often worsens substantially by the second and third decades of life, leading to severe visual impairment or legal blindness [6,13]. Currently, there is no effective therapy to prevent or reverse the retinal degeneration associated with BBS [4,8].

Despite increasing recognition of BBS as a syndromic ciliopathy, the full ophthalmologic spectrum remains insufficiently defined. Most clinical reports focus narrowly on rod–cone dystrophy, often neglecting the broader constellation of ocular abnormalities—including refractive errors, strabismus, nystagmus, optic disc pallor, early cataracts, glaucoma, and macular edema—that significantly impact long-term visual outcomes [8,13,15].

This review addresses these gaps by providing an updated and integrative synthesis of both major and minor ophthalmologic manifestations, while also highlighting recent advances in experimental therapies that may inform future clinical translation. By integrating molecular mechanisms, clinical phenotypes, and translational innovations, we aim to support precision medicine approaches and guide future research priorities in syndromic retinal disease.

## 2. Methodology

This review followed a structured literature search to synthesize current knowledge on the ophthalmologic manifestations of BBS and emerging therapeutic approaches. The process involved defining the research scope, performing database searches, applying inclusion/exclusion criteria, and organizing the findings thematically. AI-powered tools including EndNote, Grammarly, ChatGPT, Open Evidence, and Perplexity were used to assist with the literature management, thematic synthesis, citation tracking, and language editing.

### 2.1. Database Selection and Search Strategy

Peer-reviewed articles and publicly available clinical trial records were identified using PubMed, Scopus, Web of Science, Google Scholar, and official clinical trial registries (e.g., ClinicalTrials.gov). The literature search was limited to studies published between January 1995 and May 2024, and only English-language articles were included in the final synthesis. The literature search was conducted using Boolean operators (AND, OR) and included combinations of terms such as “Bardet–Biedl Syndrome,” “retinal degeneration,” “rod–cone dystrophy,” “ocular manifestations,” “gene therapy,” “ciliopathy,” “CRISPR”, and “neuroprotective treatment.” Relevant studies describing ophthalmologic manifestations or therapeutic strategies for BBS were prioritized.

### 2.2. Inclusion and Exclusion Criteria

Inclusion criteria were defined to ensure that selected studies were relevant and of high academic quality. Articles were included if they met the criteria below:

- Focused on ophthalmologic findings in patients with molecularly or clinically confirmed BBS;
- Addressed genotype–phenotype correlations or underlying genetic mechanisms relevant to ocular pathology in BBS;
- Described therapeutic interventions targeting visual outcomes in BBS.

Studies were excluded if they met the criteria below:

- Did not specifically address ophthalmologic manifestations or therapeutic approaches related to visual outcomes in BBS;
- Lacked clinical applicability or focused exclusively on non-ocular systemic features of BBS.

### 2.3. Data Extraction and Thematic Synthesis

Eligible articles were reviewed for relevant information on ocular phenotypes, disease progression, genotype associations, and therapeutic interventions. To minimize bias, two independent reviewers conducted the screening and data extraction process in duplicate, with discrepancies resolved through discussion and consensus. Findings were grouped into thematic categories: (1) retinal pathology and photoreceptor dysfunction; (2) additional ocular features; (3) emerging therapies targeting visual outcomes; and (4) current standards for ophthalmologic evaluation and management in BBS. AI tools were used to facilitate reference management and synthesis. The final manuscript integrates both molecular and clinical perspectives to provide a comprehensive overview of visual pathology and treatment advances in BBS.

## 3. Pathogenesis, Pathophysiology, and Genetics of Bardet–Biedl Syndrome

Retinal dystrophy is the most consistent and vision-limiting manifestation of BBS, arising from fundamental defects in ciliary structure and function that disrupt photoreceptor maintenance and survival [8,16,17]. In photoreceptor cells, the primary cilium forms the connecting bridge between the inner segment (IS) and outer segment (OS), acting as a specialized conduit for the bidirectional transport of phototransduction proteins from their site of synthesis in the IS to their functional location in the OS [8,16].

BBS genes are directly involved in cilium biogenesis and function, with BBS1 and BBS10 being the most frequently mutated in affected patients [17]. Seven core BBS proteins (BBS1, 2, 4, 5, 7, 8, and 9) form the BBSome complex, which mediates vesicular trafficking to the ciliary membrane, while a chaperonin-like complex composed of BBS6 (MKKS), BBS10, and BBS12 facilitates its assembly [18]. The BBSome cooperates with the IFT system to ensure proper delivery and turnover of phototransduction proteins within the cilium [19,20]. Mutations in these genes disrupt trafficking, leading to protein mislocalization, ciliary congestion, oxidative stress, and ultimately, apoptotic photoreceptor death [21]. In addition to impairing protein trafficking, BBS-related ciliary dysfunction also disrupts major cellular signaling pathways regulated by the primary cilium, such as Hedgehog, Wnt, Notch, and mTOR, which may contribute to photoreceptor degeneration and broader ocular abnormalities [3,4,16,21]. 

Histopathologic and ultrastructural studies confirm that the connecting cilium functions as a selective gate, and its disruption results in the accumulation of phototransduction proteins or exclusion of non-resident proteins, culminating in OS disorganization and retinal degeneration [16,20]. Clinically, this manifests as a rod–cone dystrophy, where rod photoreceptors are affected early, followed by progressive cone loss affecting central acuity and color discrimination [17].

While the exact roles of BBS proteins in photoreceptor cells are still not completely understood, several studies have started to shed light on how different gene mutations may lead to retinal degeneration. Mutations in BBSome components (e.g., BBS1 and BBS4) impair the directional transport of key ciliary membrane proteins such as rhodopsin and syntaxin-3, resulting in their abnormal accumulation in the IS and a reduced number in the OS [22]. This disrupts protein polarity and contributes to photoreceptor instability. 

A study conducted in BBS1-deficient zebrafish revealed that *BBS1* loss destabilizes the BBSome and leads to accumulation of membrane-associated proteins in the OS, particularly those involved in lipid homeostasis [22]. These alterations are accompanied by elevated unesterified cholesterol levels in the OS and precede morphological abnormalities and functional visual deficits, suggesting a role for *BBS1* in maintaining OS membrane integrity [22]. Further, knock-out studies in BBS5−/− mice showed a complete loss of cone function and mislocalization of cone-specific proteins such as M- and S-opsins, arrestin-4, CNGA3, and GNAT2, highlighting that *BBS5* is essential for subtype-specific protein trafficking and cone photoreceptor survival [23,24].

The severity and timing of degeneration also vary by genotype: patients with the *BBS10* mutation typically develop earlier-onset and more rapid decline compared to those with the *BBS1* mutation, as shown in both clinical and experimental models [25,26]. In *BBS10* knock-out mice, cone electroretinography (ERG) responses are absent by postnatal day 30, and rod responses decline rapidly, resulting in near-complete visual loss by nine months [26]. Structural studies in these mice have demonstrated early OS disorganization and mislocalization of cone proteins such as GNAT2 and OPN1MW by postnatal day 15 [26]. These genotype–phenotype correlations have important clinical implications as patients with *BBS10* mutations tend to experience more rapid visual decline, whereas those with *BBS1* mutations often exhibit milder and later-onset disease, supporting the utility of genetic stratification in prognostic counseling and individualized ophthalmologic surveillance.

Genotype–phenotype correlations also suggest that mutations affecting the chaperonin complex (e.g., *BBS10* and *BBS12*) result in more severe retinal phenotypes due to failure to form the BBSome complex altogether [18]. For instance, experimental models of *BBS10* and *BBS12* loss demonstrate upregulation of endoplasmic reticulum stress markers, sustained activation of the unfolded protein response, and early apoptosis of photoreceptors mediated by caspase-12 [18]. In contrast, mutations in BBSome components (e.g., BBS1, BBS4, and BBS5) allow for partial BBSome complex formation but impair its function. Notably, some BBSome components may have disproportionately critical roles, with mutations in *BBS3* and *BBS8* leading to more severe degeneration than *BBS2* or *BBS5* in murine models [21]. Although rod–cone dystrophy remains the hallmark of BBS-related retinal disease, these findings underscore that other ocular features—such as optic nerve pallor, strabismus, nystagmus, and high refractive errors—likely arise from broader defects in ciliary signaling during eye development and neuro-ocular integration [17,20].

## 4. Ophthalmologic Manifestations

### 4.1. Retinal Degeneration

Retinal degeneration is the earliest and most consistent feature of patients with BBS, affecting nearly all individuals diagnosed with the condition [17]. BBS is recognized as the second most common syndromic inherited retinal disease after Usher syndrome [17]. The characteristic degeneration closely resembles a rod–cone pattern as seen in retinitis pigmentosa (RP), where rod photoreceptors degenerate first, followed by cone loss [27,28]. Patients typically present in childhood with nyctalopia and progressive peripheral visual field constriction [29,30]. However, unlike many isolated RP cases, early involvement of the macula is common in BBS, leading to concurrent central vision loss and reduced visual acuity at a young age [11,28]. This early cone-rich macular involvement significantly impairs central vision early in the disease course [12,28]. This characteristic macular involvement has important clinical implications as it supports the need for routine macular assessment in pediatric BBS patients and the early initiation of low-vision rehabilitation to preserve functional vision and optimize developmental outcomes [11,28,29].

Ophthalmoscopic findings in the early stages often reveal a salt-and-pepper pigmentary retinopathy. As the disease progresses, optic disc pallor, attenuation of the retinal vessels, and pigment clumping with bone–spicule pigmentation develop [11,12]. Notably, the macula is affected early in BBS: subtle foveal retinal pigment epithelium mottling or a bull’s-eye maculopathy may be evident [31,32]. One retrospective study that investigated the retinal features of 46 patients with BBS revealed that 95% exhibit markedly attenuated arterioles, 84% show diffuse pigmentary retinal alterations, and 55% display bone spicule pigmentation in the mid-periphery [32]. Electroretinography (ERG) in patients with this syndrome typically demonstrates markedly reduced or extinguished rod responses in early childhood, with progressive cone dysfunction, resulting in a flat ERG by the second decade of life [30,33]. These changes correlate clinically with profound visual impairment; the majority of patients with BBS are legally blind by adulthood [29,30]. 

Although retinal degeneration is a hallmark across all genotypes of BBS, several studies have described genotype–phenotype correlations that may help clinicians predict prognosis. For instance, patients with mutations in BBS1, the most common genotype, have a milder disease course, with a later onset of symptoms and slower decline in visual acuity compared to other subtypes [11,12]. By contrast, patients with mutations in BBS10 have an earlier onset of nyctalopia, more severe reductions in the visual field, and an earlier loss of ERG responses—often by mid-childhood [11,12]. A comparative study of patients with mutations in BBS1 and BBS10 confirmed that ERG abnormalities and a loss of measurable retinal function occur significantly earlier in patients with the mutation in BBS10 [12], supporting a genotype-specific pattern of progression. 

Nonetheless, there is considerable clinical heterogeneity, even among individuals carrying identical pathogenic variants. Patients with the same BBS gene mutation may display divergent patterns of central and peripheral vision loss, suggesting the influence of modifier genes, environmental exposures, or epigenetic factors [28,34]. This heterogeneity may also be influenced by triallelic inheritance patterns, where mutations at a second BBS locus can modify the phenotypic expression of a primary biallelic mutation [35]. For example, studies have shown that the presence of a third mutant allele in BBS6 or BBS2 may worsen the clinical phenotype in patients with primary BBS1 mutations, supporting an oligogenic model of inheritance in BBS [35]. Emerging research suggests that environmental exposures, epigenetic modifications, and the presence of modifier genes may partially explain the heterogeneity in visual outcomes observed among patients with identical BBS mutations, highlighting the need for personalized ophthalmologic surveillance and prognostic counseling [28,34]. In other words, although genotype may provide insight into disease severity, it is not a definitive predictor of visual prognosis, and close, individualized ophthalmologic surveillance remains essential regardless of genetic background [11,28].

### 4.2. Refractive Errors and Corneal Abnormalities

Accumulating evidence supports that the primary cilium plays a critical role in corneal development and maintenance. It regulates signaling pathways essential for epithelial stratification, stromal organization, and corneal transparency during morphogenesis [36]. Moreover, ciliary dysfunction has been implicated in the pathogenesis of corneal disease, including curvature abnormalities and altered corneal homeostasis findings that are particularly relevant in ciliopathies such as BBS [36].

A cross-sectional, retrospective study involving 45 patients with genetically confirmed BBS (mean age 16 years) measured spherical and cylindrical refractive errors and corneal curvature [37]. The study demonstrated a strong association between the syndrome and high corneal astigmatism, with a mean astigmatism of 3.7 ± 1.0 diopters (D) and over half of the cohort exhibiting ≥ 3 D, exceeding the threshold for clinically significant astigmatism [37].

In addition to astigmatism, myopia, hyperopia, and even emmetropia have all been reported among patients with BBS, underscoring the spectrum of refractive profiles. Importantly, several studies have identified genotype–phenotype correlations in refractive outcomes. For example, individuals with BBS1 mutations exhibited a mix of myopia and hyperopia, while those with BBS10 mutations were predominantly myopic with significantly higher rates of myopia compared to BBS1 [38]. Genotypic influences have also been noted in patients with mutations in BBS3 and BBS4, further supporting the role of genetic variation in shaping ocular phenotypes in patients with BBS [39].

Astigmatism was frequently observed across genotypic subgroups and often exceeded 2.0 D, reinforcing its role as a clinically relevant feature of BBS-associated ocular pathology [38]. Given the underlying retinal dysfunction that characterizes BBS, most notably rod–cone dystrophy, early correction of refractive errors is essential. Spectacles or contact lenses should be prescribed promptly to maximize residual visual acuity, particularly in pediatric patients at risk for amblyopia or irreversible visual decline. Although randomized trials in BBS are lacking, retrospective studies and expert consensus support early and aggressive correction of refractive errors—particularly in children—as a strategy to delay amblyopia and maximize visual development during the critical period of cortical plasticity [37,40].

### 4.3. Strabismus (Ocular Misalignment)

Strabismus has also been described as an ophthalmic finding in patients diagnosed with this syndrome [41,42]. One cohort study found a 26% prevalence of strabismus and nystagmus in patients with BBS [41]. The same study reported that strabismus in these patients manifested as an even distribution between esotropias (inward turning) and exotropias (outward turning) [41]. Nonetheless, another study focusing on pediatric patients with BBS found that approximately 38% had some form of strabismus present at the initial ophthalmologic evaluation [42]—the study noted the greater prevalence of exotropia over esotropia. Due to early vision loss, strabismus in patients with this syndrome has been described as sensory in origin [40].

### 4.4. Nystagmus

Approximately 10% of patients with BBS have nystagmus, particularly those with early, profound retinal degeneration and severe visual dysfunction [40,43]. However, recent studies suggest a higher prevalence; Milibari and co-workers reported that 37% of patients with BBS presented with nystagmus as an initial ophthalmologic sign [14].

A genotype–phenotype correlation has also been described, with higher rates observed among those carrying severe variants. In a German cohort, 70% of patients with mutations in *BBS10* have nystagmus compared to 27% of those with mutations in *BBS1* [44]. Clinically, nystagmus often appears in early childhood and is typically accompanied by other visual disturbances such as night blindness, photophobia, and peripheral visual field loss [45]. Thus, the presence of early-onset nystagmus in a child with syndromic features should prompt evaluation for BBS as it may reflect significant retinal dysfunction requiring genetic and ophthalmologic assessment [40].

### 4.5. Cataracts

Cataracts are another ocular complication that have been well described in the literature in patients with BBS [46,47]. It has been described that cataracts tend to develop in patients with BBS during early adulthood [46,47]. Additionally, one study showed that genetic variability may influence the age at which cataracts appear, with a mean age of presentation of 18 years in patients with mutations in *BBS10* compared to 27 years in patients with mutations in *BBS1* [46]. Nasser and co-workers reported that 52% of patients with BBS had cataracts, and many required cataract surgeries to improve visual acuity [44]. In contrast, one pediatric-centered case series described cataracts in only a minority of patients, suggesting that cataract development in BBS may correlate with age and progression of retinal degeneration [48].

### 4.6. Optic Nerve Abnormalities

Optic nerve abnormalities are a frequently reported ophthalmologic feature in BBS, often characterized by optic disc pallor, atrophy, and narrowing of the retinal arterioles, particularly as the retinal degeneration progresses. These changes are frequently observed during ophthalmoscopic evaluation and are associated with significant visual impairment. These have been consistently reported in patients with the syndrome having advanced stages of retinal disease [17].

A recent German cohort study described that most patients with mutations in *BBS1* and *BBS10* had pale optic discs and macular atrophy [44]. Traditionally, these optic nerve findings have been considered secondary to the outer retinal degeneration; however, some evidence suggests that primary optic neuropathy may also occur. In a study by Iannaccone and co-workers, patients with BBS had early optic disc pallor in the presence of structurally preserved maculae, indicating that optic nerve atrophy may, in certain cases, represent a primary manifestation rather than a downstream consequence of photoreceptor degeneration [49]. Supporting this, advanced spectral-domain optical coherence tomography (SD-OCT) has demonstrated retinal nerve fiber layer (RNFL) thinning, while corneal confocal microscopy has revealed a loss of corneal nerve fibers in patients with BBS—findings that extend beyond photoreceptor involvement and suggest broader neuro-ophthalmologic compromise [50]. Advanced imaging modalities—particularly spectral-domain optical coherence tomography (SD-OCT) and retinal nerve fiber layer (RNFL) analysis—are increasingly recommended in clinical practice to detect early neuro-ophthalmic compromise and to guide longitudinal monitoring in patients with BBS [49,50]. Additionally, although uncommon, structural anomalies such as optic disc drusen and chorioretinal colobomas have been reported in the literature [51,52].

## 5. Emerging Therapeutic Approaches for Ophthalmologic Complications of BBS

### 5.1. Gene Therapy

As stated by the American Society of Gene and Cell Therapy, gene therapy is an excellent method to treat and prevent diseases [53]. This therapy targets the underlying genetic cause of a disease through the delivery of genetic material, in the form of DNA or RNA, to alter how a protein is produced by a cell [53]. This new genetic material that is incorporated into the cell can be delivered through a vector, often viruses, that can be administered either ex vivo or in vivo [53]. Ex vivo gene therapy involves removing cells from the patient, modifying them outside the body, and then reintroducing them into the patient [53]. In contrast, in vivo gene therapy involves directly delivering the genetic material into the patient’s body, often through injection [53].

Gene therapy offers a promising approach to addressing the genetic defects causing photoreceptor dysfunction and degeneration in the retina of patients with this syndrome. Early proof-of-concept studies have demonstrated that gene augmentation could effectively target retinal degeneration in BBS animal models. A 2011 study showed that subretinal delivery of the *BBS4* gene via an adeno-associated virus (AAV) in *BBS4*-null mice corrected rhodopsin mislocalization, improved photoreceptor outer segment structure, and preserved rod function as confirmed by electroretinogram analysis [54]. Another impactful study was published in 2013, using a knock-in mouse model carrying the common M390R mutation in the *BBS1* gene [55]. Researchers delivered the wild-type *BBS1* gene via an AAV vector through subretinal injection. This intervention partially restored BBS1 protein expression, corrected mislocalized phototransduction proteins, and preserved retinal architecture, resulting in modest but meaningful functional improvements [55].

This 2013 study also revealed a key challenge of gene therapy in patients with BBS—the potential dose-dependent toxicity that the BBS1 protein may cause. Wild-type mice (with no BBS1 deficiency) treated with the wild-type *BBS1* gene developed retinal degeneration [55]. These findings highlight the need to control transgene expression levels to avoid potential overexpression toxicity. In addition to dose-dependent toxicity, translational barriers include immune responses to AAV capsid proteins and the potential for intraocular inflammation following subretinal delivery, which could limit treatment efficacy or preclude redosing [16]. Furthermore, long-term safety remains a critical concern as persistent expression of the transgene may lead to delayed adverse effects, necessitating extended follow-up periods in future clinical trials [16,55,56]. A recent review from the Strasbourg IGMA/CIMERA group, led by H. Dollfus, elaborates on this dose-optimization challenge and details their translational AAV-BBS1 process aimed at mitigating overexpression risks [16].

More recently, a preclinical study focused on *BBS10*, a gene responsible for approximately 21% of cases, has further advanced the field of gene therapy in BBS [57]. In this study, a viral construct carrying the wild-type *BBS10* sequence was delivered subretinally in mouse models to treat retinal degeneration. Results showed that the therapy slowed photoreceptor cell death, preserved retinal structure, and delayed vision loss in the treated eyes. Importantly, cone photoreceptors, which are typically non-functional early in BBS10-related disease, regained measurable electrical function following treatment [57]. These findings are particularly encouraging as they suggest gene therapy could not only halt but also partially reverse cone dysfunction if administered early. The study also reported improvements in visually guided behavior, indicating that gene therapy preserved meaningful visual capacity over time.

Recently, a naturally-occurring *BBS7* mutation was identified in a colony of rhesus macaques, making it the first non-human primate model of BBS [58]. These monkeys exhibited classical BBS features, including progressive retinal degeneration, which Dr. Martha Neuringer and co-workers are currently testing with a subretinal gene therapy [59]. Treated eyes showed slowed degeneration and improved cone-mediated function compared to untreated eyes [59]. Building upon these preclinical successes, the first human clinical trial targeting retinal degeneration in BBS is currently in development; AXV-101 is an investigational gene therapy designed specifically for patients with the missense *BBS1* M390R mutation, the most common genetic cause of BBS [60]. This therapy uses an AAV9 vector to deliver a codon-optimized *BBS1* gene directly to the subretinal space, aiming to preserve photoreceptor cells and slow retinal degeneration. Preclinical studies have demonstrated that AXV-101 halts photoreceptor and outer nuclear retinal layer degeneration in a dose-dependent manner in BBS1M390R animal models. This therapy has shown efficacy and safety; therefore, it is expected to enter clinical trials in 2025 [60]. In addition to AXV-101, preclinical efforts are underway to develop gene therapy vectors for other common subtypes, such as BBS10 and BBS4, although clinical trials have not yet been initiated [52]. Furthermore, alternative delivery approaches, including intravitreal injection, are being explored in other inherited retinal diseases and may offer less invasive options for broader patient access in the future, although subretinal injection remains the preferred route for photoreceptor targeting in BBS at present [56,57,61].

### 5.2. Gene-Editing Therapies

CRISPR-Cas9 is a powerful gene-editing technology derived from a bacterial immune system that enables targeted modifications to DNA with high precision [62]. It uses a guide RNA to direct the Cas9 nuclease to a specific sequence in the genome, where it creates a double-strand break, allowing the DNA to be disrupted, deleted, or corrected through cellular repair mechanisms [63]. This method has revolutionized molecular biology by enabling researchers to edit genes in living organisms with relative ease, and it holds great promise for treating genetic diseases, including inherited retinal disorders like BBS [64].

Gene-editing therapies such as CRISPR/Cas9 are, therefore, being actively studied to correct the diverse pathogenic variants identified across more than 21 BBS genes [56]. Molinari, Ahmad, and co-workers state that the eye is an ideal organ for in vivo editing because of its surgical accessibility and compartmentalized immune system [61,65]. Further, Kenny and co-workers further emphasize subretinal delivery as a practical first target by keeping the gene-editing machinery confined to the eye and limiting unwanted editing elsewhere in the body [56]. Additionally, this therapy’s effect is easy to measure because any benefit or harm can be tracked with standard vision tests [56]. They also stress that successful translation will require rigorous off-target profiling, careful timing relative to disease progression, and deep-phenotyping pipelines to stratify candidates for personalized intervention [56]. Preclinical evidence supports these findings by showing how CRISPR/Cas9 disruption of the BBS modifier gene Ccdc28b in mice prevented retinal degeneration and obesity but unexpectedly produced autism-like behaviors, emphasizing the complexity of gene–gene interactions in ciliopathies and the value of genome editing for uncovering subtle phenotypic effects [66].

BBS-specific CRISPR therapies remain in the preclinical stage of research. On the other hand, patients with Leber congenital amaurosis due to the CEP290 mutation (LCA10) have been well studied in the clinical trial EDIT-101, where the first in vivo CRISPR therapy has been successfully conducted [67]. This clinical trial is extremely important because it demonstrated a favorable safety profile and visual improvement in early assessments, validating CRISPR as an alternative for subretinal delivery in humans. Sundaresan and co-workers further emphasized how the eye’s unique anatomy, accessibility, and immune privilege work as ideal conditions for safe and effective gene editing [68]. They reviewed CRISPR applications for ocular diseases, noting its precision and potential durability when correcting causal mutations in photoreceptors or retinal pigment epithelial cells. Altay and co-workers highlight that CRISPR derivatives such as base and prime editors provide greater specificity and fewer off-target effects for retinal gene editing in BBS [69]. The review by Jo and co-workers reinforces this by showing in-animal models of retinitis pigmentosa and Leber congenital amaurosis that these editors can repair mutations without double-strand breaks, improve vision, and be delivered successfully via split-AAV or non-viral vectors, a possible approach that could likewise correct many single mutations in BBS genes [69,70].

Currently, no clinical trials of gene editing have been initiated for BBS or other retinal ciliopathies; all available data are derived from preclinical animal models or in vitro systems, with the notable exception of EDIT-101 that serves as an important precedent for in vivo retinal gene editing in humans [67]. While gene editing offers considerable potential for mutation-specific correction, all current CRISPR-based therapies for BBS remain in the preclinical stage, and rigorous off-target profiling, as well as long-term safety assessments, will be essential prerequisites before initiating human trials.

### 5.3. Pharmacologic Approach—Nonsense Suppression Therapy

One targeted approach currently under investigation is nonsense suppression therapy, also known as readthrough therapy. This strategy aims to treat genetic mutations that introduce premature stop codons by enabling the cellular translation machinery to bypass the stop signal and produce a full-length, functional protein [71]. Nonsense mutations account for approximately 11% of disease-causing variants in BBS, leading to truncated and non-functional ciliary proteins that contribute to retinal degeneration [72,73]. Several pharmacologic agents have demonstrated readthrough activity in cell and animal models of other genetic disorders, including cystic fibrosis and Duchenne’s muscular dystrophy [74,75,76]. Additionally, newer translational readthrough-inducing drugs (TRIDs) have been explored in choroideremia, Usher syndrome, and retinitis pigmentosa type 2 [77,78,79]. In the context of BBS, Eintracht and co-workers conducted the first study using TRIDs in patients with BBS-derived cells [73]. Fibroblasts from a patient with *BBS2* nonsense mutations treated with ataluren or amlexanox showed restored production of the full-length BBS2 protein to approximately 35–40% of normal levels [73]. These drugs were also able to rescue ciliogenesis and cellular function in fibroblasts, suggesting that nonsense suppression therapy may hold promise for restoring protein expression and ciliary activity in cells of patients with BBS [73].

### 5.4. Antioxidant and Neuroprotective Therapies

The retinal degeneration observed in patients with BBS has been linked to increased oxidative stress, which is hypothesized to contribute to disease progression [80]. Elevated levels of mitochondrial fluorescent flavoproteins have been detected in the retinas of patients with BBS. It serves as a biomarker of oxidative stress [80]. Antioxidant therapies have therefore been investigated as a potential strategy to mitigate photoreceptor degeneration. In a *BBS10* knock-out mouse model, oral administration of N-acetylcysteine (NAC) preserved retinal structure and function [81]. Treated mice showed significantly thicker outer nuclear layers, improved electroretinogram (ERG) b-wave amplitudes, enhanced photoreceptor synaptic connectivity, and reduced oxidative stress markers compared to untreated controls [81].

Neuroprotective therapies are interventions designed to preserve neuronal integrity, prevent apoptosis, and maintain the functional capacity of cells under disease-related stress. These therapies are particularly important for patients with the syndrome because they can potentially mitigate photoreceptor death caused by ciliary dysfunction, thereby preserving vision [8,82]. One class of neuroprotective agents includes chemical chaperones, which reduce endoplasmic reticulum (ER) stress and protein misfolding, two contributors to photoreceptor apoptosis [83]. In *BBS1^M390R/M390R^* knock-in mice, the bile acid derivative tauroursodeoxycholic acid (TUDCA) preserved photoreceptor OS and maintained ERG responses compared to untreated controls [83]. TUDCA-treated mice also avoided obesity, another phenotype of the model, highlighting both retinal and systemic benefits [83]. Notably, both the NAC and TUDCA findings are derived from murine models of BBS, and although they demonstrate structural and functional preservation in photoreceptors, no human clinical trials have been conducted to date, underscoring the need for further translational validation before clinical application.

Another emerging neuroprotective treatment for patients with BBS involves the DNA damage response (DDR). Barabino and co-workers found that retinal progenitor cells and cone photoreceptors derived from a patient with the BBS10 mutation exhibited persistent DDR activation through the ATM/ATR-Chk2 checkpoint pathway [84]. This stress response contributed to early photoreceptor degeneration. Treatment with a Chk2 kinase inhibitor significantly improved tissue lamination, cone survival, and OS maturation in patient-derived retinal organoids, supporting the role of DDR modulation as a therapeutic avenue in BBS-associated retinal dystrophy [84]. Despite encouraging preclinical results, no human trials have yet been initiated for these pharmacologic or neuroprotective therapies in BBS, and translation into clinical practice will require thorough evaluation of efficacy, optimal dosing, safety, and long-term effects in prospective studies.

## 6. Current Standard Ophthalmologic Work-Up and Management in Patients with BBS

### 6.1. Ophthalmologic Work-Up of Patients with BBS

Patients with BBS require age-specific and longitudinal ophthalmologic assessments due to the progressive nature of their retinal and ocular manifestations. In infants and young children, initial evaluation should include screening for strabismus and nystagmus, along with an assessment of visual acuity using age-appropriate methods such as preferential looking or Teller acuity cards in preverbal children, and Snellen charts in older children [17,40]. In adults, visual acuity is evaluated using Snellen charts, and formal cataract assessment with slit-lamp examination is recommended [17]. Visual fields are assessed with Goldmann kinetic perimetry, tailored to the patient’s age and degree of remaining vision [40]. Cycloplegic refraction, conducted according to age-specific guidelines, is essential for determining best corrected visual acuity (BCVA) [40]. Given the high prevalence of refractive errors in patients with BBS, regular follow-up refractions are advised, and full optical correction should be prescribed unless visual function is no longer detectable [40]. Fundus photography is useful for evaluating retinal changes and optic nerve appearance and is often feasible even in younger patients [40]. Advanced imaging modalities such as optical coherence tomography (OCT) can detect outer retinal thinning and photoreceptor loss in patients who can maintain fixation, while fundus autofluorescence (FAF) helps assess retinal pigment epithelium damage, often showing a peri-macular hyper autofluorescence ring as a marker of active degeneration [40]. Follow-up care in these patients is life-long and multidisciplinary. Expert consensus recommends that children and adults with progressive disease undergo annual ophthalmologic evaluations, including visual acuity, cycloplegic refraction, OCT, and where possible, Goldmann visual field testing and FAF. In contrast, children and adults with stable visual findings may undergo follow-up every two years, although routine monitoring in adults for cataract progression, low-vision needs, and psychosocial adjustment should remain integral to care [17,40].

### 6.2. Management of Retinal Degeneration in BBS

As there is currently no available therapy that can stop the retinal degeneration in patients with BBS, clinical management is therefore supportive. Current treatment focuses on low-vision rehabilitation and adaptive strategies [40]. Patients are provided with low-vision aids (magnifiers and electronic devices) to maximize use of residual vision [40]. Orientation and mobility training are introduced early, and Braille instruction and other adaptive living skills are encouraged early at diagnosis to prepare for eventual visual deterioration [40]. Assistive tools such as white canes, guide dogs, large-print materials, and voice-recognition software can further help maintain independence as vision loss progresses [40].

Early referral to multidisciplinary support services is essential, particularly for pediatric patients, to facilitate visual development, educational access, and psychosocial adjustment [17,40]. Integration of low-vision specialists, occupational therapists, special education professionals, and mental health providers can substantially improve quality of life and functional outcomes [17,40]. Ongoing collaboration between ophthalmology and other allied fields is critical in managing progressive visual decline and in tailoring care to each patient’s evolving needs.

### 6.3. Management of Other Ophthalmologic Manifestations in BBS

Effective attention and care of other ocular issues in patients with BBS (strabismus, nystagmus, refractive errors, and cataracts) requires a supportive, interdisciplinary approach. While there is no specific cure for the sensory nystagmus in BBS, standard treatments for strabismus are applied—this includes correcting any refractive error with glasses and performing strabismus surgery when needed to improve ocular alignment and binocular function [40]. Refractive errors are extremely common and are managed with appropriate spectacles or contact lenses to optimize visual acuity [40]. Tinted eyeglasses with photoselective filters may be prescribed to reduce photophobia in these patients [40]. Additionally, vision therapy or the use of low-vision aids may be considered to help manage nystagmus-related visual instability and improve functional vision. When cataracts become visually significant, lens extraction with intraocular lens implantation is performed to improve the remaining vision [40]. However, in patients with BBS, visual outcomes following cataract surgery are highly variable and depend largely on the degree of pre-existing retinal degeneration. While some patients may experience improved contrast sensitivity or modest gains in visual acuity, others may not benefit substantially due to advanced photoreceptor loss [17,49]. Moreover, postoperative counseling should emphasize that while cataract removal may improve clarity, it does not alter the course of retinal disease, and visual expectations should be tailored accordingly.

## 7. Discussion

This review highlights that retinal degeneration is among the most consistent and earliest clinical features in patients with BBS. Notably, the pattern of degeneration often involves early macular involvement, which distinguishes BBS from other inherited retinal disorders such as nonsyndromic retinitis pigmentosa. As a result, central vision tends to be compromised at an early stage, affecting functional vision and contributing to more severe visual impairment in these patients. This hallmark macular involvement supports the rationale for routine macular imaging in pediatric patients and underscores the need for early initiation of low-vision rehabilitation to optimize developmental and educational outcomes.

Another important aspect of BBS is the genotype–phenotype correlations. Studies have shown that mutations in BBS10 are typically associated with an earlier onset and more rapid progression of visual impairment compared to those with a mutation in BBS1. Therefore, identifying the specific genetic variant is crucial during diagnosis as it can offer valuable prognostic and therapeutic information for the optimal management of these patients. In clinical practice, these correlations support individualized prognostic counseling and guide the frequency and intensity of ophthalmologic follow-up, with BBS10 patients requiring closer monitoring and earlier implementation of low-vision strategies. Still, the literature also emphasizes the marked clinical heterogeneity observed even among patients with the same mutation, suggesting that non-genetic factors may influence phenotypic expression. Despite this variability, there is general agreement that most patients with BBS present with visual dysfunction in early childhood and reach legal blindness by early adulthood. Because of this severe visual impairment during early life and the fact that visual manifestations often precede the diagnosis of other systemic features, ophthalmologists play a key role in early detection and timely referral for multidisciplinary assessment and genetic evaluation.

Even though much of the current literature focuses on retinal degeneration in patients with BBS, additional ophthalmologic manifestations—such as high refractive errors, strabismus, nystagmus, optic nerve abnormalities, and early cataracts—carry significant clinical implications. These findings can further worsen visual acuity if not identified and managed early. For instance, high astigmatism and early-onset myopia or hyperopia, when left uncorrected, may exacerbate visual decline, making timely refractive evaluation and correction essential. Additionally, features such as sensory strabismus, refractive errors, nystagmus, photophobia, and nyctalopia have been described as some of the earliest clinical signs of BBS. This reinforces that these manifestations are not incidental but rather integral components of the syndrome’s ophthalmologic profile, warranting early recognition and management by ophthalmologists. Identifying and addressing these secondary findings early is crucial because early intervention can help preserve some visual function, even when there is ongoing progressive retinal degeneration.

From a therapeutic point of view, this review illustrates how BBS has evolved from a condition with no treatment options to one with several promising proof-of-concept interventions aimed at preserving vision. Gene therapy studies targeting mutations in BBS1, BBS4, and BBS10 have shown encouraging results in animal models, demonstrating improved photoreceptor function and preservation of retinal structures. Preclinical models have shown that subretinal delivery of AAV vectors can restore BBS protein expression and correct mislocalized phototransduction proteins, delaying vision loss. However, these studies also highlight important challenges—such as vector delivery limitations and overexpression toxicity—that emphasize the need for precise control of transgene expression before moving on to human trials. Other key translational barriers include immune responses to AAV capsids, variability in dosing tolerability, and the limited predictive value of animal models for human efficacy and safety. Furthermore, current gene therapy candidates that are mutation-specific, such as AXV-101 for the BBS1 M390R variant, limit their broad applicability across the genetically diverse BBS population.

Gene-editing therapies like CRISPR-Cas9 offer a complementary strategy by targeting specific disease-causing mutations at the molecular level. Although gene editing is still in preclinical stages for BBS and no human trials have yet been initiated, early successes in related retinal disorders and the emergence of high-precision tools like base and prime editors make this therapy a promising one for the near future. Despite all the challenges faced, the recent development of a non-human primate model carrying the mutation in BBS7 represents a critical step in the development of therapeutic approaches in a more clinically relevant system.

Besides gene-based therapies, pharmacologic interventions—including nonsense suppression therapy, antioxidant strategies, and DDR pathway modulation—provide an alternative opportunity for those patients that may not qualify for gene-based therapies. Nonsense suppression therapy can be of particular interest for patients with premature stop codons on the BBS protein because these therapies have shown a way to restore full-length protein synthesis. For example, preclinical studies with therapies like ataluren and amlexanox highlight this therapeutic potential, although clinical translation will require validation of efficacy and durability in vivo. In addition to mutation-targeted approaches, therapies aimed at protecting photoreceptors from secondary damage have been studied. Rather than targeting the genetic defect, antioxidant and neuroprotective strategies work by ameliorating cellular damage that contributes to retinal degeneration caused by oxidative stress, protein misfolding, or DNA instability. Such therapies offer a different treatment pathway that can be of therapeutic use across the genetically diverse mutations causing BBS. By having a different mechanism of action, these pharmacologic interventions could be used as adjunctive treatments to gene-based therapies. This offers a distinct path of visual preservation even when genetic mutations cannot be corrected.

However, the systemic delivery of these agents poses important challenges for targeting the retina. The blood–retinal barrier significantly limits the intraocular availability of systemically administered compounds, potentially reducing therapeutic efficacy. In contrast, intraocular administration—such as intravitreal or subretinal injection—may achieve higher local concentrations but involves procedural risks and does not address systemic BBS manifestations. Nonsense suppression agents like aminoglycosides have shown potential for restoring protein synthesis in preclinical models but carry known risks of retinal and cochlear toxicity when delivered systemically or locally at high doses. Similarly, while antioxidants such as N-acetylcysteine have shown retinal benefits in BBS10–/– mouse models, no clinical trials have confirmed their ocular safety or long-term tolerability in humans. These limitations underscore the need for careful consideration of the delivery route, dosing, and toxicity profiles when advancing systemic or intraocular therapies for BBS-related retinal degeneration.

Despite significant advances, many therapeutic approaches for BBS remain in the experimental or preclinical stages. Emphasizing the importance of supportive ophthalmologic care, such as timely refractive correction, low-vision rehabilitation, and cataract surgery, remains the standard of clinical management. However, the field is rapidly evolving, and ophthalmologists are essential for connecting experimental therapies with clinical care through early diagnosis, patient stratification, and involvement in ongoing therapy trials. Such approaches represent encouraging steps toward preserving vision in this patient population.

## 8. Conclusions

This review emphasizes the importance of early diagnosis and the need for a comprehensive ophthalmologic evaluation in patients with BBS. One of the most significant symptoms of BBS is the early onset of retinal degeneration, which leads to vision loss and disabilities. Other ophthalmologic complications may further compromise vision if not properly recognized and evaluated in a timely manner. While recent advancements in gene-based therapies and pharmacologic strategies show great potential, it is important to note that no disease-modifying treatments for BBS are currently approved, and supportive care remains the standard of management. By implementing genetic testing alongside personalized ophthalmologic care, we can significantly improve patient morbidity and health outcomes.

Recent advancements in gene-based therapies, gene editing, and pharmacologic approaches may provide various methods for preserving visual health and could become standard care for these patients. The current understanding of the pathophysiology and molecular mechanisms of BBS is still evolving, effective therapeutic options remain limited, and significant challenges persist in translating research into clinical practice. As our knowledge of this syndrome continues to grow, the outlook for patients with BBS becomes increasingly hopeful, bringing us closer to effective and potentially transformative treatments.

## 9. Future Directions

Future directions for BBS research should prioritize the translation of current preclinical studies into safe and effective treatment options for patients with BBS. Gene augmentation shows promise; however, it requires safe and effective delivery methods to minimize toxicity. Major challenges include AAV vector size limitations, achieving efficient transduction of photoreceptors, and minimizing immune responses and off-target effects. The same applies to gene editing—we need to further understand how to deliver it in vivo and assess its long-term effects. Pharmacologic approaches still need to be adequately validated to understand their true potential as most studies remain in the preclinical stages. Future research should involve more patients and active collaboration with ophthalmologists to report ocular findings. This will help build a more complete picture of the ocular manifestations of BBS and ultimately allow us to better co-manage these patients. Future research should explore alternative therapies, such as cell transplantation and retinal prosthetic devices in patients with BBS.
